# Effectiveness of Punch Grafting in Promoting Healing and Reducing Pain in Hard‐to‐Heal Leg Ulcers

**DOI:** 10.1111/wrr.70126

**Published:** 2026-01-13

**Authors:** Julia Neuenschwander, Dieter O. Mayer, Ramon Lang, Caroline Staub‐Buset, Mirjam Nägeli, Jürg Hafner

**Affiliations:** ^1^ Department of Dermatology University Hospital Zurich Zurich Switzerland

**Keywords:** chronic leg ulcer, hard‐to‐heal leg ulcer, punch graft, punch grafting, recalcitrant leg ulcer, skin grafting

## Abstract

Approximately 20% of chronic leg ulcers remain recalcitrant despite treatment of underlying factors and best standard of wound care. This study aimed to evaluate the effectiveness of partial‐thickness punch grafting in promoting healing and reducing pain in patients with chronic, hard‐to‐heal leg ulcers of various causes. In this single‐centre, retrospective cohort study, 93 patients were treated between January 2016 and December 2024, with follow‐up at 1, 3, 6 and 12 months. The primary outcome was complete wound healing, while secondary outcomes included pain reduction and wound surface changes. At 12 months, 78/88 analysed patients (88.6%) achieved complete healing of the target ulcer. Pain levels improved substantially, with the proportion of pain‐free patients increasing from 17.6% at baseline to 76.3% at 6 months. Donor site complications were minimal (6.5%) and cosmetic outcomes were excellent. Recurrence after 12‐month follow‐up occurred in only 9% of healed ulcers within 12 months. These results confirm that partial‐thickness punch grafting is a highly effective and minimally invasive technique for treating hard‐to‐heal leg ulcers, delivering durable healing, rapid pain relief and low morbidity. This study provides new long‐term data supporting the broad clinical utility of punch grafting across diverse ulcer types.

## 1 Introduction

Chronic ulcers, particularly, those affecting the lower limbs, impose a significant burden on healthcare systems worldwide [1, 2, 3, 4, 5, 6, 7]. These ulcerations impact up to 49 million people annually, with a lifetime risk of 1.0%–1.8%, increasing with age [8, 9, 10, 11]. In the United States alone, the treatment of chronic wounds costs an estimated $25 billion each year [12]. A 2024 editorial analysing wound care expenditures by the Top 10 global spenders found that 2022 estimates ranged from $6 billion to $148 billion, confirming the immense worldwide financial burden of wounds [13].

Clinical and best practice guidelines consistently highlight the importance of a comprehensive assessment for patients with chronic wounds [14, 15, 16, 17, 18]. Impaired healing is typically the result of multiple systemic, regional, and local factors. When these underlying causes are properly addressed, approximately 80% of chronic wounds heal successfully [19]. Chronic leg ulcers that fail to reduce in size within the first four to six weeks of treatment, despite optimal management of all wound healing barriers, are classified as hard‐to‐heal (non‐healing, recalcitrant or refractory) ulcers [16, 20].

Hard‐to‐heal wounds are commonly managed through tissue transfer techniques or the application of skin substitutes. A key advantage of skin grafts is their ability to accelerate wound healing and alleviate pain by facilitating the release of growth factors from transplanted cells [21, 22]. Until recently, the standard approach for treating hard‐to‐heal wounds involved tangential debridement (necrosectomy), followed by split‐thickness skin grafting (STSG) [23]. In some cases, negative pressure wound therapy (NPWT) was utilised between debridement and grafting to enhance granulation tissue formation, promote wound bed preparation and aid in the removal of necrotic tissue and toxins [24]. These procedures, however, often require general anaesthesia and hospitalisation, contributing to the overall complexity and cost of treatment.

In recent years, the pinch graft technique, first introduced by Dr. Jacques‐Louis Reverdin in 1869 as *greffes épidermiques* [25, 26], has experienced a significant resurgence [27]. This minimally invasive procedure involves transplanting small, circular skin grafts (*greffes epidermiques en pastilles*), typically harvested from the thigh, into ulcerated areas to promote healing [28, 29]. Both pinch grafting and its more recent derivative, punch grafting, have regained increasing recognition as effective treatments for hard‐to‐heal leg ulcers [30, 31, 32, 33, 34].

The Department of Dermatology at University Hospital Zurich treats about 80 new patients with hard‐to‐heal leg ulcers annually, following international treatment guidelines [35, 36]. From 1995 to 2015, shave therapy with STSG was the gold standard for managing hard‐to‐heal leg ulcers [37]. In 2016, we introduced the less invasive partial‐thickness punch graft (PTPG) technique for outpatient ulcer repair [30]. This paper analyses patient outcomes over an 8‐year period (2016–2024), assessing the efficacy of PTPGs in complex wound management.

## 2 Patients and Methods

### 2.1 Study Design

This single‐centre, retrospective clinical‐observational study included 116 consecutive patients who received PTPGs for hard‐to‐heal leg ulcers between January 2016 and December 2024 at a tertiary university hospital.

Inclusion criteria were based on the presence of hard‐to‐heal leg ulcers as defined by the Harmonised Glossary of Wound Care Terms and consistent with the Swiss National Guidelines for the use of skin replacement procedures in hard‐to‐heal wounds [38].

Exclusion criteria were attendance at fewer than two of the four scheduled follow‐up visits, absence from the 12‐month follow‐up or receipt of a split‐thickness skin graft after the initial punch graft procedure.

### 2.2 Study Population

**FIGURE 1:**
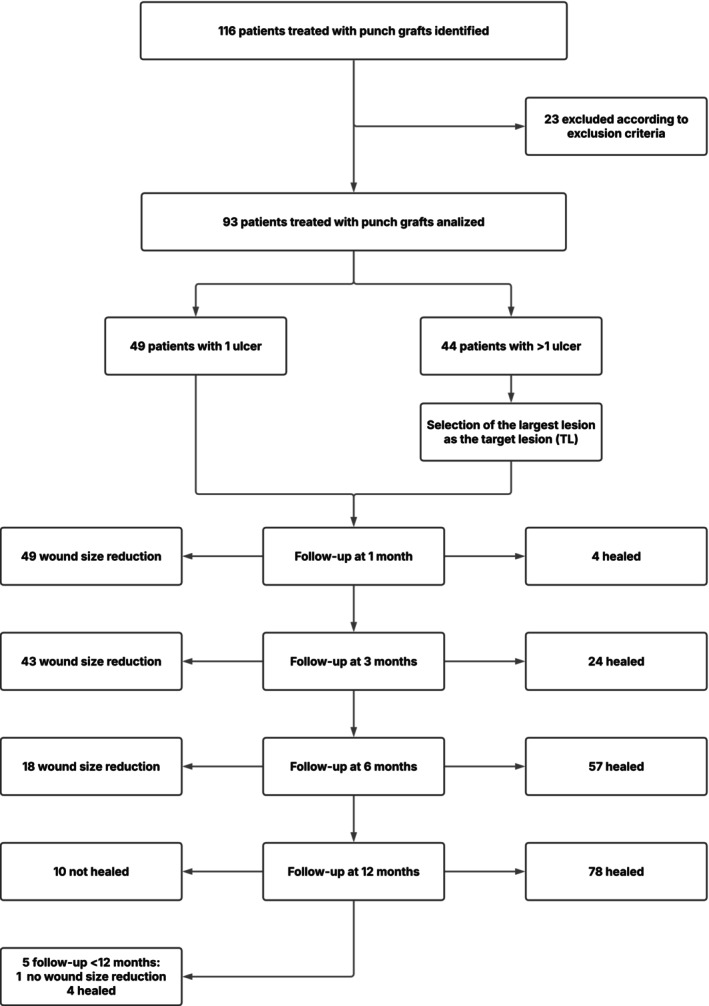
Flowchart of study design and results.

We identified all patients who underwent PTPG treatment in our Dermatology Department from January 2016 to December 2024, regardless of wound aetiology. During this 8‐year period, 116 patients received PTPGs for hard‐to‐heal leg ulcers. After applying exclusion criteria, 23 patients were excluded, leaving 93 consecutive patients for analysis (Figure 1). The median age was 72 (min 29, max 99) years and the proportion of male to female was 47/46.

Common comorbidities included arterial hypertension (68.8%), chronic venous disease (55.9%) and diabetes mellitus (24.7%). Approximately one‐third (30%) had a history of peripheral arterial disease. Klinefelter syndrome was diagnosed in two patients (2.2%). At baseline, 51 patients (54.8%) were on basic pain medication and 20 (21.5%) required opioids in addition to basic analgesic therapy. The detailed patient demographics and comorbidities are described in Table 1.

**TABLE 1:** Patient demographics and comorbidities.

	Patients (*n* = 93)
Demographics	
Male/female, *n* (%)	47 (50.5)/46 (49.5)
Age (years), mean (SD)	72.0 (13.3)
BMI (kg/m^2^), mean (SD)	28.1 (7.3)
Comorbidities and risk factors	
Arterial hypertension, *n* (%)	64 (68.8)
Smoking, *n* (%)	39 (41.9)
Current	17 (18.3)
Former	22 (23.7)
Never smoked	53 (57.0)
Chronic venous disease, *n* (%)	52 (55.9)
Peripheral arterial disease, *n* (%)	28 (30.1)
Diabetes mellitus, *n* (%)	23 (24.7)
Polyneuropathy, *n* (%)	21 (22.6)
Depression, *n* (%)	7 (7.5)
Malnutrition, *n* (%)	2 (2.2)
Klinefelter syndrome, *n* (%)	2 (2.2)
Baseline medication	
Nonsteroidal anti‐inflammatory drugs, *n* (%)	51 (54.8)
Opioids, *n* (%)	20 (21.5)
Platelet aggregation inhibitors, *n* (%)	32 (34.4)
Anticoagulation therapy, *n* (%)	32 (34.4)
Immunosuppressants, *n* (%)	8 (8.6)

Abbreviations: *n* = number; SD = standard deviation.

Tables 2 and 3 provide an overview of the hard‐to‐heal study wounds and detail the characteristics of all target lesions, respectively. In our dermatologic study population undergoing PTPGing, we identified three main patient groups: vascular, non‐vascular and atypical. Table 4 shows the characteristics of non‐healed target lesions at 12 months.

**TABLE 2:** Overview on hard‐to‐heal study wounds.

	Patients (*n* = 93)
All lesions	
Number of patients with > 1 leg ulcer, *n* (%)	44 (47.3)
Bilateral leg ulcers, *n* (%)	19 (20.4)
Total number of treated leg ulcers per patient, mean (SD)	2.0 (1.7)
Target lesion (largest ulcer treated)	
Outpatient treatment, *n* (%)	50 (53.8)
Number of punch grafts procedures, mean (SD)	1.4 (0.7)
Time between procedures (months), median (Q1–Q3)^a^	3 (2–7)
Total number of punches (*n*), median (Q1–Q3)^a^	33 (15–48)

Abbreviations: *n* = number; SD = standard deviation.

^a^ Interquartile range.

**TABLE 3:** Characteristics of the target lesion.

	Patients (*n* = 93)
Aetiology of ulcers, *n* (%)	
Vascular	54 (58.1)
Venous	32 (34.4)
Mixed (venous + arterial)	14 (15.1)
Arterial (peripheral arterial disease)	8 (8.6)
Non‐vascular	23 (24.7)
Burn	1 (1.1)
Infectious	12 (12.9)
Postoperative	10 (10.8)
Atypical	16 (17.2)
Martorell hypertensive ischemic leg ulcer	7 (7.5)
Other (detailed in results paragraph text)	9 (9.7)
Location of target lesion, *n* (%)	
Right/left lower extremity	39 (41.9) / 54 (58.1)
Calf	38 (40.9)
Malleolus medialis	19 (20.4)
Malleolus lateralis	17 (18.3)
Foot	16 (17.2)
Thigh	3 (3.3)
Wound size at baseline (cm^2^), mean (SD)	8.4 (9.2)
Wound size at baseline (cm^2^), median (Q1–Q3)^a^	5.6 (2.6–10.9)
Wound persistence (months), median (Q1–Q3)^a^	6 (3–12)
Wound infection, *n* (%)	46 (49.5)
Previous split‐thickness skin graft, *n* (%)	21 (22.6)

Abbreviations: *n* = number; SD = standard deviation.

^a^ Interquartile range.

**TABLE 4:** Non‐healed target lesion characteristics.

Non‐healed target lesions at 12 months	*n* = 10
Wound persistence (months), median (Q1–Q3)^a^	8 (4–17)
Aetiology of ulcers, *n* (%)	
Vascular	10 (100)
Venous	5 (50)
Mixed (venous + arterial)	3 (30)
Arterial (peripheral arterial disease)	2 (20)
Non‐vascular	0 (0)
Burn	0 (0)
Infectious	0 (0)
Postoperative	0 (0)
Atypical	0 (0)
Martorell hypertensive ischemic leg ulcer	0 (0)
Other (detailed in results paragraph text)	0 (0)
Baseline wound surface (cm^2^), mean (SD)	7.0 (5.2)
Wound surface after 1 month (cm^2^), mean (SD)	5.5 (4.4)
Wound surface after 3 months (cm^2^), mean (SD)	4.7 (6.0)
Wound surface after 6 months (cm^2^), mean (SD)	4.4 (6.4)
Wound surface after 12 months (cm^2^), mean (SD)	10.5 (11.6)
Number of punch grafts procedures, mean (SD)	1.8 (1.2)

Abbreviations: *n* = number; SD = standard deviation.

^a^ Interquartile range.

### 2.3 Surgical Procedure

PTPG procedures were performed either during hospitalisation or as outpatient procedures, depending on the patient's physical and mental condition.

A potent anaesthetic cream (lidocaine 23%, tetracaine 7%) was applied to the chronic wound for 20 min while punch grafts were harvested from the ipsilateral thigh. The donor site, approximately 1.5 times larger than the wound, was anaesthetised with 0.5% lidocaine. Using a 5‐ or 6‐mm punch, grafts were pre‐incised to the mid‐dermis, with 2–200 grafts harvested depending on wound size. The PTPGs were harvested at a thickness of approximately 0.4–0.5 mm. The biopsy punch was advanced into the dermis with a gentle twisting motion, stopping just before reaching the subcutaneous fat. Afterwards, the grafts were released by a horizontal incision, usually with a scalpel or surgical forceps. This creates a circular donor‐site defect measuring 6 mm in diameter (or corresponding to the punch size) with a white dermal wound bed. The yellow subcutaneous tissue is not exposed. Each PTPG was then placed on moist gauze. The donor site was dressed with chlorhexidine acetate‐impregnated paraffin gauze, cotton wool gauze and non‐woven retention tape.

Debridement with a 7‐mm ring curette was then performed with minimal pain. PTPGs were spaced ~5 mm apart on the wound and covered with a silicone non‐adhesive gauze, calcium alginate dressing, cotton wool gauze and a non‐woven retention tape. A four‐layer bandage was applied in all patients except in patients with arterial leg ulcers.

### 2.4 Additional Procedures

Histology was performed on 43 patients (46.2%), while microbiological smears were obtained from 72 patients (77.4%).

### 2.5 Data Collection

Data were retrospectively extracted and anonymized from electronic medical records (KISIM, CISTEC AG, Zurich, Switzerland) in compliance with Good Clinical Practice (GCP). Collected data included patient demographics, comorbidities, medications, pain levels, wound aetiology and duration, baseline characteristics, microbiology results from wound swabs, number of punch grafting procedures and grafts applied, clinical photographs and wound surface planimetry measurements recorded before the punch graft procedure and during visits.

Patients whose wound status could not be assessed through medical records or visits at any time were contacted by phone.

### 2.6 Follow‐Up

After the procedure, dressings were changed every 3–4 days for two weeks. During the first month, a dermatologist conducted weekly evaluations, with follow‐ups adjusted based on wound healing progress.

Per protocol (Figure 1), mid‐term to long‐term follow‐ups were scheduled at 3, 6 and 12 months postoperatively. All but five patients attended the 12‐month follow‐up visit; four had healed and one remained unhealed. These five were excluded from the final 12‐month outcome analysis. Patients who completed the 12‐month visit were subsequently followed either clinically or by telephone for an additional period of up to 12 months.

### 2.7 Outcome Analysis

The primary objective was to evaluate the wound closure or complete healing rate, with wound surface reduction and pain reduction as secondary endpoints.

In nearly half of the patients, multiple ulcers were treated. The largest ulcer was designated as the target lesion and if treated multiple times, healing time was measured from the first punch graft intervention.

Pain was categorised as none, mild, moderate or severe, but its assessment was discontinued after 6 months due to limited data.

To evaluate safety, complications such as bleeding and infection were analysed. While wound characteristics were assessed only for the target lesion, complications at both the ulcer and donor sites were recorded for all punch graft interventions.

### 2.8 Statistical Analysis

Descriptive statistics were reported as frequencies and percentages for categorical variables. Continuous variables were expressed as means and standard deviation (SD) or as median with range or interquartile range (Q1–Q3).

A Pearson's *χ* ^2^ test was performed to test for statistical differences between categorical variables with two‐sided *p* < 0.05 considered significant.

Statistical analyses and charts were performed using IBM SPSS Statistics, Version 30.0 (IBM Corp. Armonk, NY).

### 2.9 Ethics

The local Ethical Committee approved the study protocol in March 2021 (BASEC ID 2021‐00621), in accordance with the 1975 Declaration of Helsinki. Individual patient data are available upon request, pending ethical approval. All participants provided written informed consent.

### 2.10 Definitions

A hard‐to‐heal wound is a wound that has failed to progress through the phases of healing in an orderly and timely fashion and has shown no significant progress towards healing in 30 days (i.e., chronic wound or non‐healing wound) [38].

The target lesion is defined as the ulcer treated in patients with a single ulcer and the largest ulcer treated in those with multiple ulcers.

Wound closure or complete healing, was defined as full re‐epithelialization of the wound surface with no further requirement for wound dressings, in accordance with the guidelines of the European Wound Management Association (EWMA) [39].

Wound recurrence is defined as the reopening or breakdown of a previously healed wound at the same anatomical site following punch grafting.

## 3 Results

In our cohort, nearly half of the patients (47.3%) had multiple ulcers, with 19 (20.4%) having ulcers on both legs (Table 1). Outpatient care was provided to 50 patients (53.8%), with an average of 1.4 (SD 0.7) procedures per target lesion and a median interval of 3 months (IQR 2–7) between interventions. The median number of punches used per target lesion was 33 (IQR 15–48), as shown in Table 2.

Table 3 presents the main characteristics of the target lesions. The study categorised the target lesions into three main groups: vascular, non‐vascular and atypical. Vascular ulcers were the most common, affecting 54 patients (58.1%), with venous ulcers being the predominant type within this category (34.4%). The non‐vascular and atypical ulcer groups contained 23 (24.7%) and 16 (17.2%) patients, respectively. Among the non‐vascular ulcers, the most frequent causes were infectious (12.9%) and postoperative (10.8%). The atypical group consisted of 16 patients, with hypertensive ischemic leg ulcers (Martorell's ulcer) being the most common cause, accounting for seven cases (44% of this subgroup). Vasculitis and scleroderma were the next most frequent aetiology with two cases (13% each). Livedoid vasculopathy, cryoglobulinemia, dystrophic calcification, mycosis fungoides and pyoderma gangraenosum accounted for the remaining cases (6.3% each).

Most ulcers were located on the calf (40.9%), followed by the medial malleolus (20.4%) and lateral malleolus (18.3%). The mean baseline wound size was 8 cm^2^ (SD 9.2), with a median wound duration of 6 months (IQR 3–12). Infection was identified in 46 patients (49.5%), either before or during the intervention, with *Pseudomonas aeruginosa* and *Staphylococcus aureus* as the most common pathogens. Prior to the PTPG procedure, 21 patients (22.6%) had already undergone a STSG.

Within 12 months, wound healing was achieved in 78/88 patients (88.6%), with a mean healing time of 6.2 months (SD 3.8). The target lesion was healed in 4, 24, 57 and 78 patients at the 1‐, 3‐, 6‐ and 12‐month follow‐ups, respectively (Figure 1).

**FIGURE 2:**
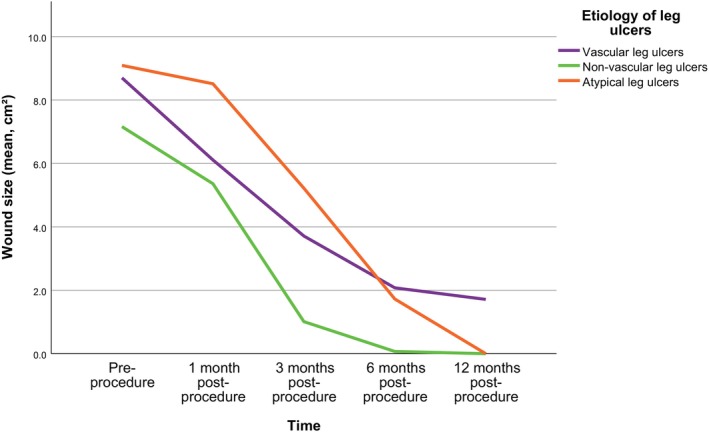
Aetiology‐based timeline of wound size reduction after initial punch grafting. Non‐vascular and atypical wounds exhibited a steeper healing trajectory from 3 to 12 months post‐procedure compared with vascular wounds. By 12 months, all non‐vascular and atypical wounds had completely healed, whereas 10 vascular wounds remained unhealed at this time point.

Following the PTPG procedure, the mean wound surface area for all wounds decreased significantly from 8.4 cm^2^ (SD 9.2) at baseline to 0.9 cm^2^ (SD 4.5) by 12 months. During this period, non‐vascular wounds exhibited the fastest healing trajectory, with complete closure of all wounds. Wounds of atypical aetiology showed an initially delayed healing response during the first month, but subsequently followed a similarly steep healing curve, also resulting in full wound closure at 12 months. In contrast, vascular wounds demonstrated a much flatter healing curve, with 10 wounds failing to heal (Figure 2, Table 4). Table 5 summarises wound closure rates and recurrence by aetiology. At 6 months, non‐vascular ulcers demonstrated the highest healing rate (85.7%), followed by atypical ulcers (68.8%) and vascular ulcers (54.9%). At this time point, non‐vascular wounds healed significantly better than vascular wounds (*p* = 0.013), while no statistically significant differences were observed between the other groups.

**TABLE 5:** Healed ulcers and recurrences according to aetiology.

Aetiology	Healed at 6 months, *n* (%)	Healed at 12 months, *n* (%)	Recurrence after 12 months, *n* (%)
Vascular ulcers (*n* = 51)	28 (54.9)	41 (80.4)	5 (9.3)
Venous (*n* = 30)	18 (60.0)	25 (83.3)	4 (12.5)
Mixed (venous + arterial) (*n* = 13)	5 (38.5)	10 (76.9)	1 (7.1)
Arterial (peripheral arterial disease) (*n* = 8)	5 (62.5)	6 (75.0)	0 (0)
Non‐vascular ulcers (*n* = 21)	18 (85.7)	21 (100.0)	1 (4.8)
Burn (*n* = 1)	1 (100.0)	1 (100.0)	0 (0)
Infectious (*n* = 12)	9 (75.0)	12 (100.0)	1 (8.3)
Postoperative (*n* = 8)	8 (100.0)	8 (100.0)	0 (0)
Atypical ulcers (*n* = 16)	11 (68.8)	16 (100.0)	1 (6.3)
Martorell hypertensive ischemic leg ulcer (*n* = 7)	5 (71.4)	7 (100.0)	0 (0)
Other (*n* = 9)	6 (66.7)	9 (100.0)	1 (11.1)
Vasculitis (*n* = 2)	2 (100)	2 (100.0)	0 (0)
Livedoid vasculopathy (*n* = 1)	0 (0)	1 (100.0)	1 (100.0)
Scleroderma (*n* = 2)	1 (50.0)	2 (100.0)	0 (0)
Cryoglobulinemia (*n* = 1)	1 (100.0)	1 (100.0)	0 (0)
Dystrophic calcification (*n* = 1)	1 (100.0)	1 (100.0)	0 (0)
Mycosis fungoides (*n* = 1)	1 (100.0)	1 (100.0)	0 (0)
Pyoderma gangraenosum (*n* = 1)	0 (0)	1 (100.0)	0 (0)

Abbreviation: *n* = number.

By 12 months, all non‐vascular and all atypical ulcers had achieved complete healing, whereas 41 of 51 vascular ulcers (80.4%) were healed. The same pattern persisted at 12 months, with non‐vascular wounds again healing significantly better than vascular wounds (*p* = 0.021) and no significant differences between the remaining group comparisons. At 12 months follow‐up, 10 patients (11.4%) had persistent open wounds (Table 4). All of these patients (100%) had vascular ulcers. Their mean wound size increased from 7 cm^2^ (SD 5.2) before the intervention to 10.5 cm^2^ (SD 11.6) at 12 months. In this group, the median ulcer duration before punch grafting was 8 months (IQR 4–17), with an average of 1.9 grafting procedures per target lesion. Notably, two of these ulcers healed after the 12‐month follow‐up visit.

Donor site complications were observed in six patients (6.5%). Minor bleeding occurred in four patients (4.3%), while infection was noted in one patient and another developed eczema (1.1%).

Target lesion complications occurred in 21 patients (22.6%) following split‐thickness punch grafting, with infection being the most common in 19 patients (20.4%). Two patients (2.2%) developed wound eczema.

Two patients suffering from infections ultimately required below‐knee amputation. The first patient, with critical illness polyneuropathy, underwent PTPG for an open wound on the big toe. However, the ulcer failed to heal, became infected and progressed to osteomyelitis, necessitating below‐knee amputation. The second patient had pre‐existing osteomyelitis caused by *beta‐hemolytic group G streptococci*. Despite bone resections, flap plasty and STSG‐ing, the wound remained open. Two PTPG procedures were attempted, but due to progressive osteomyelitis, below‐knee amputation was ultimately required.

The overall recurrence rate after the 12‐month follow‐up was 9% (7/78). Recurrence was highest among vascular ulcers (9.3%), followed by atypical ulcers (6.3%) and non‐vascular ulcers (4.8%). Within the vascular group, venous ulcers showed a recurrence rate of 12.5%, while mixed arterial–venous ulcers recurred in 7.1% of cases. In comparison, non‐vascular and atypical ulcers exhibited recurrence rates of 4.8% and 6.3%, respectively. The median ulcer duration before the initial PTPG procedure was 5 months (IQR 4–48), which is similar to the overall cohort median of 6 months (IQR 3–12). Among the seven recurrent cases, one patient underwent four PTPG procedures, while another received PTPG three times. The complication rate for recurrent ulcers was 28.6%, with infection occurring in both affected patients. Before the PTPG intervention, 31 patients (34.1%) reported moderate pain, 23 (25.3%) experienced severe pain, 21 (23.1%) had mild pain and 16 (17.6%) reported no pain. One month post‐intervention, the number of pain‐free patients increased to 48 (55.2%), while 22 (25.3%) reported mild pain, 13 (14.9%) moderate pain and 4 (4.6%) severe pain. By 6 months, 61 patients (76.3%) were pain‐free, 10 (12.5%) had mild pain and 9 (11.2%) continued to experience moderate to severe pain.

## 4 Discussion

Hard‐to‐heal ulcers or wounds, though lacking a universally precise time‐based definition, are typically characterised by the absence of healing progress or worsening condition within 4–6 weeks after initiating appropriate local, regional, or systemic treatment, based on a thorough diagnostic evaluation [16, 17, 20]. These wounds often remain stalled in the inflammatory phase of healing and are frequently the result of disrupted interactions (known as ‘dynamic reciprocity’) between key cell types (such as fibroblasts, keratinocytes and inflammatory cells that release growth factors and cytokines) and the surrounding extracellular matrix [40]. Without interruption of this vicious cycle through advanced treatment methods, healing is unlikely to occur [41].

In our cohort of 93 patients, median leg ulcer persistence was 6 months (IQ 3–12), despite adequate treatment of the cause and the local wounds following international standards (Table 3). The complexity of this cohort was underscored by the fact that 23% of patients had previously undergone at least one split‐thickness skin graft procedure without achieving a successful outcome.

Following standardised PTPG treatment, early outcomes at 6 months showed that nonvascular ulcers achieved significantly higher healing rates than vascular ulcers (86% vs. 55%, respectively; *p* = 0.013). In contrast, the difference between non‐vascular and atypical ulcers (86% vs. 69%, respectively; *p* = 0.214) did not reach statistical significance.

By 12 months, the overall healing rate in this hard‐to‐heal leg ulcer cohort had reached 89%. Healing outcomes were comparable between the 41 inpatients and the 47 outpatients (*p* = 0.818). At this time point, complete closure was observed in all non‐vascular and all atypical ulcers, whereas 80% of vascular ulcers had healed. In the aetiology‐based comparison, non‐vascular wounds again showed superior healing compared with vascular wounds (*p* = 0.021), with no other significant differences detected between groups.

As illustrated in Figure 2, the three wound categories exhibited distinctly different healing dynamics. Non‐vascular ulcers followed a comparatively steep healing curve, with the majority already healed by 6 months. Vascular ulcers demonstrated a more linear trajectory during the first 6 months, after which their curve flattened and remained so until 12 months postoperatively; notably, 10 ulcers in this category did not heal. In contrast, atypical ulcers showed a slow initial phase with a relatively flat curve during the first 3 months, followed by a markedly steeper and more linear healing progression. Thereafter, their trajectory converged with that of the non‐vascular ulcers, with all wounds in both categories achieving complete healing. The overall cure rate observed in our study exceeds the average outcomes reported by other research groups employing punch grafting [30, 33] or pinch grafting [31, 32, 34] techniques for the treatment of hard‐to‐heal leg ulcers. Notably, in 2008, Nordström and colleagues published the first study evaluating punch grafting to accelerate healing and alleviate pain in complex leg and foot ulcers [33]. In their retrospective case series of 22 patients, 86% of whom had venous or mixed aetiology vascular ulcers, they reported a healing rate of 50% at six months. These results are in line with our own intermediate‐term findings. However, no follow‐up beyond six months was conducted, limiting comparisons at the 12‐month time point.

In a more recent retrospective, non‐comparative cohort study published in 2017, Groening and co‐authors analysed data from 213 patients with a total of 284 ulcers [30]. They reported a healing rate of 18.7% at three months post punch grafting, which increased to 52.2% at 12 months. In contrast, our study demonstrated a 12‐month healing rate of 89% in a comparable, though slightly more complex, patient population. Several methodological differences may help explain the improved outcomes observed in our cohort. Specifically, we utilised larger punch grafts (5–6 mm in diameter) compared to the 4 mm grafts used by Groening and colleagues and employed silicone non‐adhesive gauze combined with calcium alginate dressings rather than paraffin gauze and hydrofiber or foam dressings for post‐grafting wound care. Previous research has indicated that calcium alginate may promote more rapid wound healing than hydrofiber dressings [42], potentially contributing to the superior healing outcomes achieved in our study.

Pinch grafting, a technique related more closely to the originally described technique by Reverdin [25] and refined thereafter by Davis [43], seems to show less favourable results than punch grafting in our cohort. In the retrospective study conducted by Oien et al., 126 chronic wounds from 85 patients were analysed [34]. An overall healing rate of 60% was achieved within 12 months following pinch grafting. Specifically, 67% of venous ulcers and 33% of mixed vascular ulcers healed within this period. In comparison, our cohort demonstrated higher wound closure rates of 83% and 77% for the same ulcer types, respectively. A key difference between the two studies lies in the methodology: Oien et al. analysed multiple wounds per patient, whereas our study focused on a single (the largest) target wound per patient. Additionally, the time from wound onset to the initial pinch grafting intervention differed by approximately 3 months, with our study initiating PTPG treatment earlier.

In another retrospective case series, Hjerppe et al. examined 169 ulcers from 104 patients [31]. An overall healing rate of 61.5% was achieved, with outpatients healing more rapidly (median 5 weeks; range 3–12 weeks) than inpatients (median 8 weeks; range 1–44 weeks). Complete healing was observed in 62.8% of venous ulcers and 58.3% of mixed vascular ulcers, both of which represent substantially lower closure rates compared to those achieved in our cohort. It is important to note, however, that the duration of ulcers prior to initial pinch grafting intervention was considerably longer in the study by Hjerppe et al. than in ours. This observation suggests that earlier application of pinch or punch grafting in hard‐to‐heal ulcers may lead to improved outcomes. Nevertheless, systematic long‐term follow‐up after healing was not conducted in their study, making direct comparison with our cohort challenging.

More recently, two small prospective case series have investigated hair follicle grafting for hard‐to‐heal leg ulcers [44, 45]. Both studies employed the same harvesting and transplantation protocol, which differs from traditional punch and pinch grafting techniques. Hair follicle units (1‐mm circular punches) were extracted from the occipital scalp region using a micromotor device and approximately five follicular grafts per square centimetre were implanted into the wound bed using SAVA implanters. In both cohorts, a significant reduction in wound size and volume was observed within 8–18 weeks. However, due to the absence of mid‐ to long‐term follow‐up, complete wound closure rates were not reported. The small sample sizes (15 and 10 patients, with 17 and 14 ulcers, respectively) as well as differences in tissue type and harvesting techniques complicate direct comparison to our results. These findings highlight the need for larger, ideally comparative, studies to further validate the efficacy of hair follicle grafting.

### 4.1 Recurrence Rates

In our cohort of patients with hard‐to‐heal leg ulcers treated with punch grafting, recurrence rates after the 12 months follow‐up were notably low. The overall recurrence rate was just 9%, underscoring the durability of healing achieved with this approach. Recurrence was highest among vascular ulcers at 9.3%, which is expected given the underlying circulatory compromise. Even within this challenging group, venous and mixed arterial–venous ulcers had relatively modest recurrence rates of 12.5% and 7.1%, respectively. In contrast, non‐vascular and atypical ulcers demonstrated very low recurrence rates of 4.8% and 6.3%, respectively, further highlighting the effectiveness of PTPG across diverse ulcer etiologies. Notably, the previously cited studies on punch or pinch grafting do not report recurrence rates, making our findings a valuable contribution to the literature.

A relevant question for future research is whether pinch grafting yields lower recurrence rates than punch grafting, as the former technique transplants a greater portion of the dermis, which may confer more durable wound closure. Addressing this hypothesis would require a prospective randomised controlled trial. Additionally, tangential ablation of fibrotic tissue, commonly employed in ‘shave surgery’ [46, 47], may also play a role in reducing recurrence rates of hard‐to‐heal venous leg ulcers (VLUs). However, this method is not typically combined with the minimally invasive punch grafting technique and its potential benefit in this context remains to be determined.

### 4.2 Complications

**FIGURE 3:**
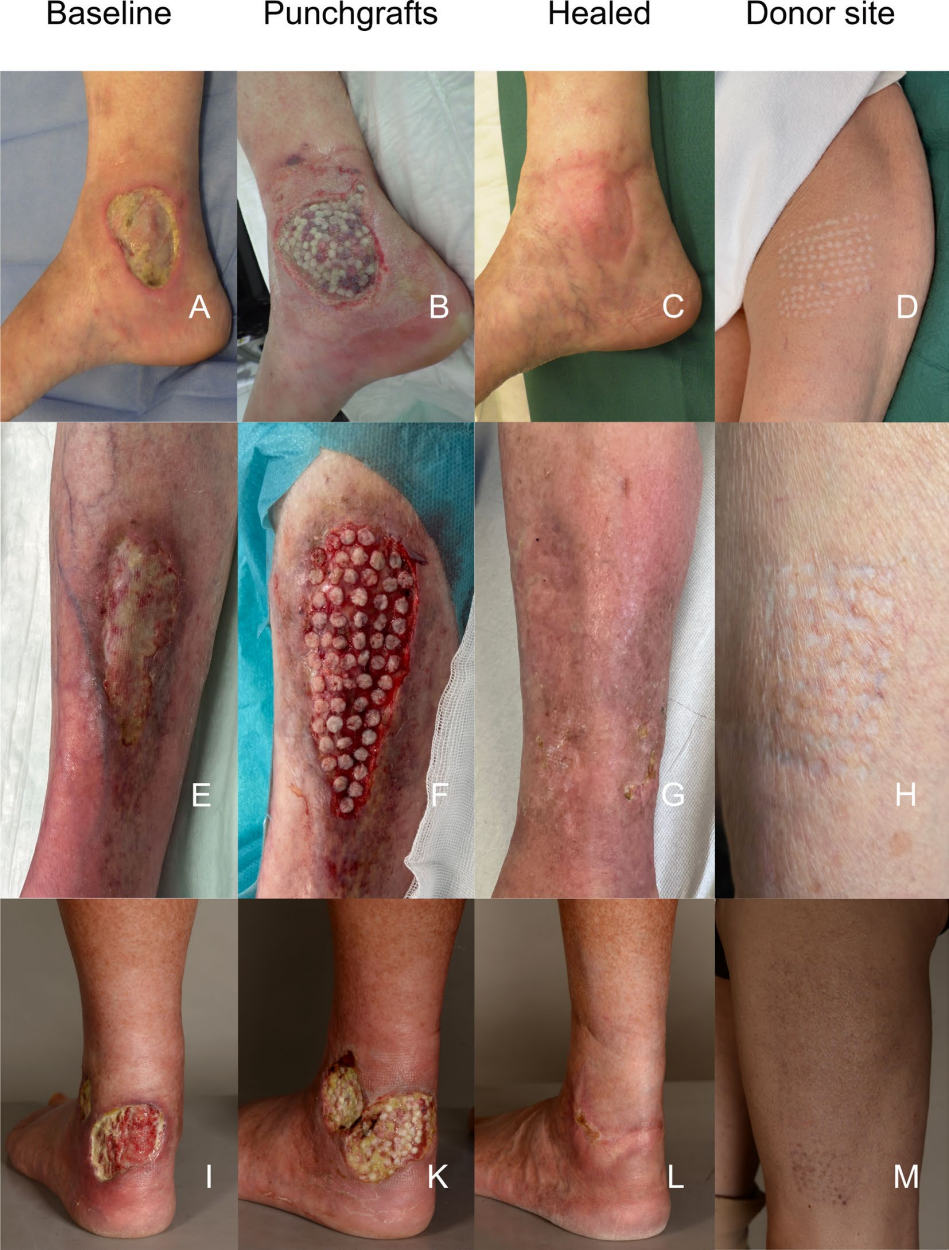
Ulcers treated with punch grafting technique. The first row of images depicts the wounds at baseline, the second row shows the wounds shortly after punch grafting, and the third and fourth rows display the healed wounds and donor sites, respectively. (A–D) A 77‐year‐old female patient with a venous ulcer over the right medial malleolus, present for 5 months. The ulcer was treated in a single session with 90 punch grafts, resulting in complete healing after five months. (E–H) An 82‐year‐old female patient with a trauma‐induced ulcer on the left lower leg, persisting for three months. The ulcer was treated with a single punch graft session applying 60 punch grafts. Postoperatively, the patient developed erysipelas, with *Pantoea calida* identified as the causative agent. Complete wound healing was achieved 9 months after the intervention. (I–M) A 66‐year‐old male patient with a hypertensive ischemic leg ulcer (Martorell ulcer) on the left lower leg, present for 2 months. The ulcer was treated with punch grafting in three sessions at approximately 1.5‐month intervals, with a total of 291 punch grafts applied. Complete wound healing was achieved after 12 months. (I–M) Have been republished with the permission of the journal *Revue Médicale Suisse* [29]. *Note:* All donor sites have healed flawlessly, resulting in excellent functional and cosmetic outcomes.

In our PTPG cohort, donor sites demonstrated favourable healing outcomes, with a low overall complication rate of 6.5%. The most common issue was minor punch hole bleeding (4.3%). By one year post‐procedure, the donor sites had healed with an excellent functional and cosmetic outcome in the majority of patients (Figure 3), underscoring the minimal morbidity associated with this technique.

By comparison, donor site morbidity reported in split‐thickness skin graft studies is substantially higher. In a prospective case series of 120 patients, Otene et al. observed high complication rates at multiple time points: at 1 month, dyschromia (39.2%) and itching (22.5%) were most prevalent; by 3 months, hyperpigmentation (55.4%) and dyschromia (37.5%) were common and by 6 months, 96% of patients exhibited hyperpigmentation, with 4% developing hypertrophic scars [48].

Similarly, Bache et al. reported considerable patient‐reported donor site issues following thin and super‐thin STSG harvests [49]. Among the 107 respondents (43% response rate), 52% experienced itch, 48% pain and 24.7% reported dressing leakage. Wound breakdown occurred in 11.5%, along with complaints such as odour, tightness and oozing. Long‐term complications included pigmentation changes (32%), hypervascularity (24%), abnormal texture (19%), firmness (13%) and altered sensation (10%). Even among recipients of super‐thin grafts (0.003–0.005 in.), the complication rates remained notably higher than those seen in our punch‐graft cohort.

Efforts to mitigate STSG donor site morbidity include techniques such as regrafting with minced residual grafts [50, 51]. However, a recent systematic review by Asuku et al. highlights the persistent burden of donor site morbidity, with scar scores up to 10.9 (on a 0–13 scale), infection rates as high as 56% and long‐term hypertrophic scarring reported in up to 28% of patients at 8 years [52].

Collectively, these findings suggest that PTPG may offer significant advantages over STSG in terms of donor site healing, with lower complication rates and superior long‐term cosmetic outcomes.

Target lesion and other wound complications were observed in 21 patients (22.6%) of our cohort following PTPG treatment, with infection being the most frequent complication (20.4%). Two patients (2.2%) developed wound eczema. Notably, two infections resulted in below‐knee amputations: one due to osteomyelitis following a non‐healing ulcer in a patient with critical illness polyneuropathy, and the other from refractory, pre‐existing osteomyelitis. In a comparable large retrospective non‐comparative study examining outcomes of punch grafting for hard‐to‐heal leg ulcers, a similar recipient site infection rate of 27.2% was reported [30]. In contrast, a smaller pilot study documented a significantly lower infection rate of 4.5% [33]. Two studies on pinch grafting for hard‐to‐heal leg ulcers did not explicitly report complication or infection rates, which highlights the need for standardised reporting in future research.

### 4.3 Wound Pain

The effectiveness of the PTPG technique in reducing wound pain in our patients was particularly impressive. They reported a significant reduction in wound pain as quickly as 1–2 days after the procedure. This improvement was especially marked among patients with hypertensive ischemic leg ulcers (Martorell ulcers), who experienced pronounced pain relief immediately following skin grafting. Accompanying the pain reduction was a notable improvement in sleep quality and overall quality of life.

Our results regarding pain reduction after PTPG align closely with previous studies. In a pilot study by Fourgeaud et al. involving 42 patients, 76% initially reported daily permanent pain, and all experienced pain peaks [22]. One day after the PTPG procedure, 77% reported less permanent pain and 90% fewer pain peaks. At discharge, improvements rose to 90% and 95%, respectively. PTPG was particularly effective for hypertensive ischemic leg ulcers (Martorell ulcers), and 81% of patients reduced strong opioid analgesic use post‐procedure.

This improvement is supported by Nordström et al., who reported a significant reduction in pain scores from 4.2 to 0.8 on the Visual Analogue Scale (VAS) after the intervention [33]. Similarly, Conde‐Montero et al., in a study specifically designed to measure pain outcomes involving 136 patients with hard‐to‐heal leg ulcers, noted that 63% achieved complete pain suppression (VAS = 0) within a week [53]. Pain reduction was consistent across various ulcer types, notably in VLUs and was independent of the percentage of graft take.

### 4.4 Cost Efficiency

Our retrospective case series did not assess the cost‐efficiency of punch grafting; however, existing literature supports its economic viability. Selva‐Sevilla et al. conducted a cost‐utility and cost‐effectiveness analysis comparing punch grafting to standard care for chronic wounds [54]. Their findings indicated that punch grafting reduced treatment costs by 37% and increased wound‐free days by an average of 7.2 days within a 3‐month period.

The simplicity and affordability of punch grafting make it particularly suitable for low‐resource settings. A study in rural Cameroon involving 13 elderly patients with chronic leg ulcers demonstrated that punch grafting led to complete healing in 12 patients within 12–14 weeks, with minimal donor site complications [32]. This underscores punch grafting's feasibility and effectiveness in environments with limited medical infrastructure.

Given the substantial costs associated with chronic wound management, the adoption of cost‐effective and minimally invasive techniques like punch grafting could alleviate financial burdens on healthcare systems. Future controlled studies are warranted to further evaluate the economic benefits and long‐term outcomes of punch grafting in diverse clinical settings.

### 4.5 Limitations

The study's reliance on a retrospective and observational design, conducted at a single specialised hospital, inherently limits its ability to establish definitive causality and potentially restricts the generalizability of its findings to other settings. However, this real‐world approach enabled the evaluation of a substantial cohort of 93 patients over an eight‐year period, offering pragmatic insights into how PTPG performs in a complex clinical environment. This design also ensured robust long‐term follow‐up within the same cohort. Furthermore, while the study lacked a concurrent control group receiving alternative treatments for direct comparison, it diligently contextualised its findings by comparing them to published outcomes for various grafting methods. These comparisons indicated favourable results for PTPG, particularly highlighting lower complication rates and superior cosmetic outcomes at the donor site compared to traditional split‐thickness skin grafts, alongside referencing literature that supports its cost‐effectiveness.

The heterogeneity of wound etiologies and patient comorbidities, characteristic of a tertiary care centre, could present challenges in drawing definitive conclusions for specific ulcer types. To mitigate this, the study categorised outcomes based on whether ulcers were vascular, non‐vascular or atypical. This sub‐analysis revealed the technique's broad effectiveness, including high success rates even for challenging vascular and atypical ulcers, and its utility in cases where prior standard treatments like split‐thickness skin grafts had failed.

A methodological consideration is the detailed analysis focusing primarily on the largest wound, or target lesion, per patient. This approach might not fully encapsulate the healing trajectory for individuals presenting with multiple ulcers. Nevertheless, it allowed for consistent monitoring of the most clinically relevant wound, demonstrating an impressive 12‐month healing rate of nearly 90% and a notably low recurrence rate of under 10%; outcomes that are often not reported in comparable studies.

Finally, the retrospective nature of the study made the collection of precise, standardised pain scores challenging. Despite this, the available information strongly indicated a substantial and clinically meaningful reduction in pain reported by patients shortly after PTPG treatment, with a majority becoming pain‐free within six months. This reinforces a key benefit of the procedure and aligns with findings from other research.

## 5 Conclusion

Based on this 8‐year study of 93 patients, PTPG demonstrated significant effectiveness and multiple benefits for treating complex, hard‐to‐heal leg ulcers. An impressive 89% of target ulcers achieved complete healing within 12 months, with non‐vascular wounds healing significantly better than vascular wounds at this time point (*p* = 0.021). Patients experienced substantial and often rapid pain reduction, with the majority becoming pain‐free by 6 months, a benefit, particularly, notable for painful Martorell hypertensive ischemic leg ulcers. The treatment yielded durable results, indicated by a low overall recurrence rate of only 9% after the 12‐month follow‐up. Furthermore, the procedure involves minimal donor site morbidity, with few complications and generally excellent cosmetic outcomes compared with traditional methods such as split‐thickness skin grafts. Significant decreases in wound size were also consistently observed.

Overall, partial‐thickness punch grafting emerges from this extensive clinical experience as a highly effective and minimally invasive technique that offers high healing rates, including statistically superior outcomes for nonvascular ulcers, together with substantial pain relief, durable long‐term results and minimal donor site problems for patients with complex and hard‐to‐heal leg ulcers.

## Author Contributions

All authors made substantial contributions to the conception, design, data acquisition, analysis or interpretation of the work, has participated in drafting or revising the manuscript and has approved the final version.

## Funding

The authors have nothing to report.

## Disclosure

All listed authors meet the authorship criteria defined by the International Committee of Medical Journal Editors (ICMJE). No portion of the manuscript was developed using Artificial Intelligence–Generated Content (AIGC) tools, including ChatGPT or other large language models (LLMs).

## Conflicts of Interest

The authors declare no conflicts of interest.

## Data Availability

The data that support the findings of this study are available on request from the corresponding author. The data are not publicly available due to privacy or ethical restrictions.
